# Liposomal Delivery of Newly Identified Prophage Lysins in a *Pseudomonas aeruginosa* Model

**DOI:** 10.3390/ijms231710143

**Published:** 2022-09-04

**Authors:** Diana Morais, Luís Tanoeiro, Andreia T. Marques, Tiago Gonçalves, Aida Duarte, António Pedro Alves Matos, Joana S. Vital, Maria Eugénia Meirinhos Cruz, Manuela Colla Carvalheiro, Elsa Anes, Jorge M. B. Vítor, Maria Manuela Gaspar, Filipa F. Vale

**Affiliations:** 1 Pathogen Genome Bioinformatics and Computational Biology, Research Institute for Medicines (iMed.ULisboa), Faculty of Pharmacy, Universidade de Lisboa, 1649-003 Lisboa, Portugal; 2 Advanced Technologies for Drug Delivery, Research Institute for Medicines (iMed-ULisboa), Faculty of Pharmacy, Universidade de Lisboa, 1649-003 Lisboa, Portugal; 3 Faculty of Pharmacy, Universidade de Lisboa, Av. Gama Pinto, 1649-003 Lisboa, Portugal; 4 Centro de Investigação Interdisciplinar Egas Moniz (CiiEM), Instituto Superior Egas Moniz, Quinta da Granja, 2829-511 Monte da Caparica, Portugal; 5 Host-Pathogen Interactions Unit, Research Institute for Medicines (iMed-ULisboa), Faculty of Pharmacy, Universidade de Lisboa, 1649-003 Lisboa, Portugal

**Keywords:** *Pseudomonas aeruginosa*, prophage, bacteriophage, phage therapy, lysins, antibiotic resistance, liposomes, gram-negative bacteria

## Abstract

*Pseudomonas aeruginosa* is a Gram-negative opportunistic bacterium that presents resistance to several antibiotics, thus, representing a major threat to human and animal health. Phage-derived products, namely lysins, or peptidoglycan-hydrolyzing enzymes, can be an effective weapon against antibiotic-resistant bacteria. Whereas in Gram-positive bacteria, lysis from without is facilitated by the exposed peptidoglycan layer, this is not possible in the outer membrane-protected peptidoglycan of Gram-negative bacteria. Here, we suggest the encapsulation of lysins in liposomes as a delivery system against Gram-negative bacteria, using the model of *P. aeruginosa*. Bioinformatic analysis allowed for the identification of 38 distinct complete prophages within 66 *P. aeruginosa* genomes (16 of which newly sequenced) and led to the identification of 19 lysins of diverse sequence and function, 5 of which proceeded to wet lab analysis. The four purifiable lysins showed hydrolytic activity against Gram-positive bacterial lawns and, on zymogram assays, constituted of autoclaved *P. aeruginosa* cells. Additionally, lysins Pa7 and Pa119 combined with an outer membrane permeabilizer showed activity against *P. aeruginosa* cells. These two lysins were successfully encapsulated in DPPC:DOPE:CHEMS (molar ratio 4:4:2) liposomes with an average encapsulation efficiency of 33.33% and 32.30%, respectively. The application of the encapsulated lysins to the model *P. aeruginosa* led to a reduction in cell viability and resulted in cell lysis as observed in MTT cell viability assays and electron microscopy. In sum, we report here that prophages may be important sources of new enzybiotics, with prophage lysins showing high diversity and activity. In addition, these enzybiotics following their incorporation in liposomes were able to potentiate their antibacterial effect against the Gram-negative bacteria *P. aeruginosa*, used as the model.

## 1. Introduction

The Gram-negative bacterium *Pseudomonas aeruginosa* is found in diverse habitats. It is an important opportunistic pathogenic bacterium responsible for acute or chronic infections in humans, particularly among immunocompromised individuals, patients with cystic fibrosis, the elderly, and hospitalized patients [1,2,3]. About 9% and 7% of the healthcare-associated infections are caused by *P. aeruginosa*, in Europe and the US, respectively [4].

*P. aeruginosa* presents resistance to several antibiotic classes, namely beta-lactams, aminoglycosides, and quinolones. The underlying resistance mechanisms include intrinsic resistance (reduced outer membrane permeability, antibiotic-efflux pumps and antibiotic–inactivating enzymes), adaptive resistance (biofilm formation and point mutations), and acquired resistance (horizontal transfer of antibiotic resistance genes) [3,5]. Presently, infections by antibiotic-resistant bacteria show an increasing trend, making antibiotic resistance a global health emergency and one of the major threats to human (and animal) health. This global antibiotic crisis was recognized by the World Health Organization (WHO) that published in 2017 the first ever list of antibiotic-resistant priority pathogens for the development of new medicines, where we can find the carbapenem-resistant *P. aeruginosa* in the critical priority group [6].

Bacteriophages, or phages, are viruses that infect bacteria. Phage therapy uses phages and their lytic lysins, representing, nowadays, an alternative antibacterial class in the fight against infectious disease [7]. Phage therapies are in their 100th anniversary and their capability to kill susceptible organisms has attracted much attention as potential substitutes for conventional antibiotics [8]. The co-evolution between phages and their host bacteria for billions of years permitted the acquisition of mechanisms to counter bacterial defences. Moreover, recent animal and human trials showed phages to be safe, well-tolerated agents and highly promising as alternatives to chemical agents [9]. Phages may follow a lytic cycle, promptly leading to cell lysis after bacterium infection and phage replication or a lysogenic cycle, integrating in the host genome (named prophage) and vertically transmitted by host cell division. Prophages may switch to the lytic cycle in the presence of an inducing event. Not infrequently, in long term lysogeny, prophages became remnant, incapable of switching to a lytic cycle [10,11,12].

Lysins make part of the lysis cassette of phages, being synthetized towards the end of the phage cycle. They can hydrolyse the bacterial peptidoglycan, leading to the release of the phage progeny. The peptidoglycan is part of the bacterial cell wall, located on the outside of the cytoplasmic membranes, being formed by linear glycan strands cross-linked by short peptides. The glycan strands are formed by the chains of the disaccharide repeat *N*-acetylglucosamine and *N*-acetylmuramic acid, linked by β(1 → 4) glycosidic bonds [13]. Lysin is a generic name given to peptidoglycan hydrolysing phage enzymes, which can be classified as glycosidases, which cleave one of the two glycosidic bonds of the glycan chain, and may be subdivided in: (i) *N*-acetyl-β-D-glucosaminidases, *N*-acetyl-β-D-muramidases (or lysozymes), and lytic transglycosylases; (ii) amidases, which cleave the amide bond between the first amino acid residue of the peptide chain and the *N*-acetylmuramic acid; or (iii) peptidases, which cleave peptide bonds of the peptide strands or remove C-terminal amino acid residues (carboxypeptidases) [14,15]. Phage enzymes are the most promising for an enzybiotic (enzymatic antibiotic) application due to their very high activity and specificity and to the tremendous phage diversity available in the biosphere [16]. However, lysins are mainly used in Gram-positive bacteria, since lysins make direct contact with the cell wall when added externally, whereas the outer membrane of Gram-negative bacteria prevents this interaction. Several approaches have been used to overcome this limitation. One strategy uses membrane permeabilizers, such as ethylenediaminetetraacetic acid (EDTA), that were demonstrated to permeabilize the cell membrane of several Gram-negative bacteria without affecting the action of the endolysins under study. However, its applicability in vivo is limited, due to EDTA toxicity [17]. Other strategy involves the use of phage endolysins modified in the laboratory, such as artilysins (lysin fusion with an outer membrane destabilizing peptide) [18]. Another alternative consists in the incorporation of lysins in lipid-based systems, such as liposomes, aiming to deliver the loaded lysin to the peptidoglycan layer. Liposomes are spherical structures constituted by one or more phospholipid bilayers, separated by aqueous compartments, which allows them to incorporate hydrophilic or hydrophobic compounds [19,20]. This strategy has demonstrated an improvement to the pharmacokinetic profiles of loaded compounds and, consequently, their therapeutic activity both in vitro and in vivo [20,21,22,23]. In particular, the lipid composition chosen in the present work, composed of DPPC: DOPE and CHEMS with fusogenic properties, has already demonstrated the enhancement of the antibacterial effect of vancomycin against Gram-negative bacteria [24,25]. Here, we intend to identify putative lysin genes in prophages of *P. aeruginosa*, clone and express the lysin genes in *Escherichia coli*, and incorporate them in liposomes, with the aim of studying the lysin-mediated lysis in a *P. aeruginosa* model.

## 2. Results

### 2.1. Complete Prophages Are Common in P. aeruginosa Genomes

A total of 38 distinct complete prophages were identified in the group of *P. aeruginosa* genomes studied (Table 1). These prophages have a mean GC content of 61%, considerably less than the GC content of the sequenced strains (66%), which is typically found in prophages and is compatible with horizontal transfer [11]. According to VIRFAM [26], 30 phages were of the identified as *Siphoviridae* (*n* = 23), *Myoviridae* (*n* = 5), or *Podoviridae* (*n* = 2). For the remaining eight, no family was predicted.

**Figure 1:**
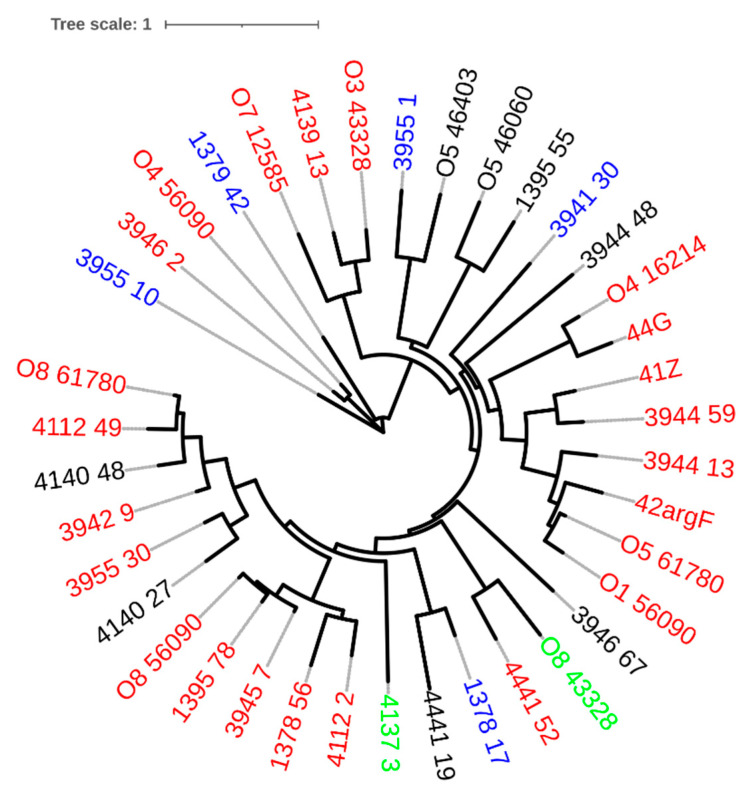
Phylogenetic tree of prophage genomic sequences, using the Jukes–Cantor substitution model and no bootstrapping/likelihood as branch support method in PHYML 3.3.20180621 (Geneious version 2021.1.1). The tree was visualized with iTOL v6 [30]. Prophages found in *P. aeruginosa* genomes retrieved from PATRIC start with capital letter O. Each tree leaf is color-coded according to the predicted phage family: red—*Siphoviridae*; blue—*Myoviride*; green—*Podoviridae*.

The genomic phylogenetic of the prophages showed that they do not cluster according to family membership (Figure 1), meaning that the predicted morphotype of the virion do not correlate with the genome phylogeny. Indeed, due to this discrepancy, the International Committee on the Taxonomy of Viruses is developing a new classification system based on genome and proteome data [29]. This group of prophages served as target sequences for the identification of lysins.

### 2.2. Pseudomonas Phage Lysins Are Functionally and Genetically Diverse 

**Figure 2:**
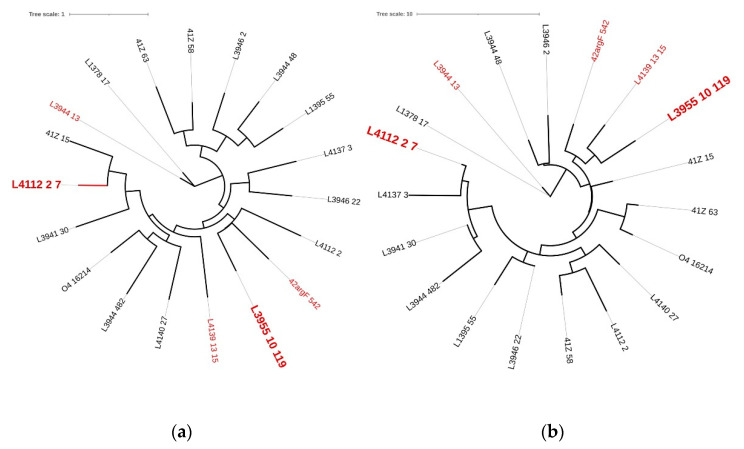
Phylogenetic trees of prophage lysins based on (**a**) nucleotides and (**b**) amino acids sequences. Genomic tree was constructed using the Jukes–Cantor substitution model and no bootstrapping/likelihood as branch support method and the proteomic tree was constructed using the Le Gascuel substitution model and no bootstrapping/likelihood as branch support method in PHYML 3.3.20180621 (Geneious version 2021.1.1). Trees were visualized using iTOL v6 [30]. Red—endolysins selected for cloning; larger leaf names—selected for liposome encapsulation.

Lysins encoded by prophage genomes are particularly interesting, since they are isolated from the target microorganism for which phage (lysin) therapy is intended [31]. Complete prophages are expected to carry a lysis cassette, including a gene coding for a lysin, so that the phage lytic cycle can be concluded. Here, we have identified 19 putative lysins (Table 2). However, we cannot discard that additional phage lysins may be present in the current dataset because three major limitations in databases that hamper the correct annotation of phage genes [32]: divergence and size of phage sequences, alignment-based annotation, and poor representation of viruses in databases. The nucleotide and amino acid phylogenetic trees were used to analyze the diversity found among this group of lysins, while the BLASTp [33], InterPro [34], and Phyre2 [35] analysis was determinant to predict the biochemical mode of action of the putative lysins. Aiming at a wide range of nucleotide and amino acid diversity (Figure 2), as well as the predicted mode of action of the putative lysins (Table 2), we have selected five lysins for cloning, hereinafter named Pa7, Pa13, Pa15, Pa119, and Pa542 (Table 3).

Lysin Pa7 showed high homology with *P. aeruginosa* phage-encoded lysozymes. Lysin Pa13 demonstrated high homology to the enzyme chitinase. Chitinases cleave the β-1,4 bonds in the chitin molecule, a polymer of *N*-acetylglucosamine. Although lysozymes and chitinases do not share a significant similarity with the protein primary sequence, they have a highly identical structure and mode of action, namely between the chitinase family 19 and the lysozymes encoded by phage T4 [36]. Lysin Pa15 had a query cover of 100% and a percent identity close to 100% with endolysins encoded by different *P. aeruginosa* phages, being identified as a transglycosylases, which cleave the glycosidic bond between *N*-acetylmuramyl and *N*-acetylglucosaminyl residues. Lysin Pa542 had identity with endopeptidases, which cleave the peptidoglycan between any of the amino acids of the cross bridges. Finally, lysin Pa119 showed high homology with muramidases encoded by *P. aeruginosa* phages (Table 3).

### 2.3. Expression and Antibacterial Activity of Phage Lysins: Putative Lysins Are Lysins de Facto

**Figure 3:**
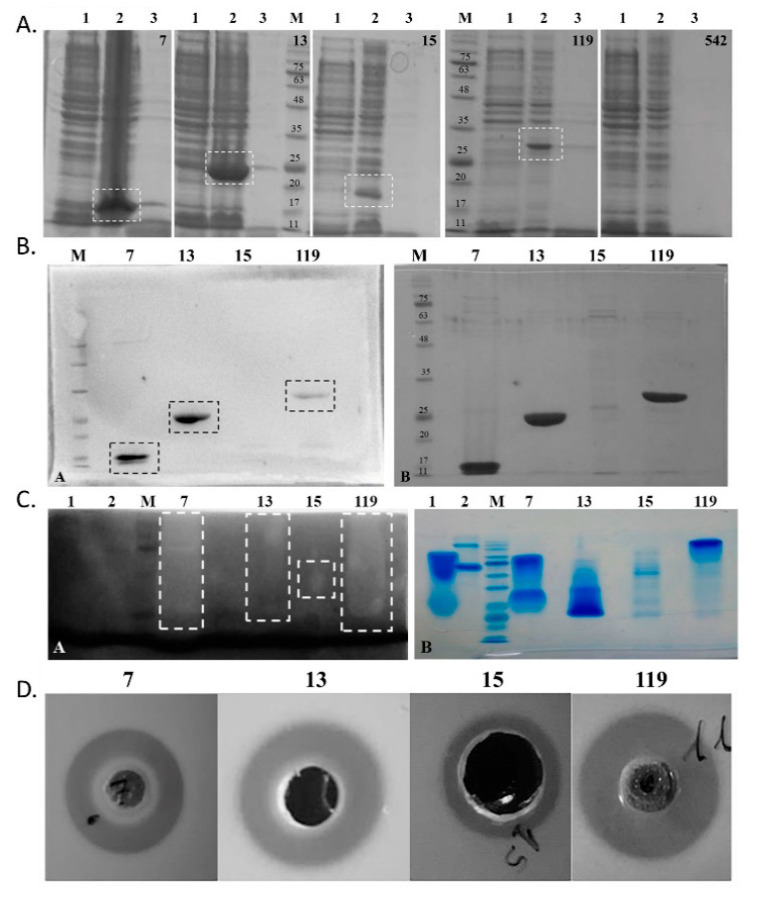
Overall assessment of purification and activity over peptidoglycan layer of phage lysins. Top right number depict phage endolysins from the left to right: 7—lysin Pa7 (expected molecular weight: 16.2 kDa); 13—lysin Pa13 (22.9 kDa); 15—lysin Pa15 (23.5 kDa); 119—lysin Pa119 (29.3 kDa); and 542 –lysin Pa542 (16.0 kDa); M—NZYColour Protein marker II (NZYTech, Lisbon, Portugal) with 75, 63, 48, 35, 25, 20, 17, and 11 kDa bands pointed out in (**A**,**B**). (**A**) SDS-PAGE of induction assays of the cloned lysins for (1) non-induced cells, (2) 3 h induced cells and (3) induced culture supernatants. Induced bands are highlighted (dotted box). (**B**) Western blot (left panel) and parallel SDS-PAGE (right panel) of the purified lysins. Identity of the produced recombinant proteins was confirmed by western-blot targeting (His)^6^-tagged proteins. Protein bands are highlighted (dotted boxes). (**C**) Zymogram using *P. aeruginosa* ATCC 27853 biomass (left panel) and parallel SDS-PAGE under zymogram conditions (right panel) of the purified lysins. Hydrolytic activity was detected as clear bands in the dark background (dotted boxes). (**D**) Halo-formation assays on *M. luteus*. Bacteriolytic activity of the purified endolysins was qualitatively assessed against *M. luteus* in LB agar plates after small-scale kit purification. Positive bacteriolytic activity appears as a clear halo around the protein application as well. For each lysins, 30 µg were applied per well. A total volume of 75 µL was applied in the wells, except for Endolysin Pa15 for which a large well was needed and a 200 µL total volume was applied.

All lysins were successfully cloned in pET15b and confirmed by Sanger sequencing. Concerning protein expression, all lysins but Pa542 were expressed, presenting molecular weights similar to the expected ones (Figure 3a and Table 3). Furthermore, the western blot targeting the (His)^6^-tag tail in each lysin confirmed the purification of the desired protein (Figure 3b). The lysin Pa542 was not expressed despite three distinct IPTG concentrations (0.1, 0.4 and 1 mM) being tested for induction. Furthermore, a comparison with a control not induced with IPTG, showed that, after IPTG induction, a decrease in the OD at 600 nm is observed, which indicates that the expression of this lysin might be toxic for *Escherichia* *coli*. Concerning Pa15, the expression was possible, but the concentration of this lysin after purification was low. Indeed, for lysin Pa15, a decrease in the OD at 600 nm was observed after IPTG induction, reducing the amount of lysin produced.

The zymogram performed using autoclaved cells of *P. aeruginosa* shows that the four lysins hydrolyse the peptidoglycan, as evidenced by a white band at around the expected position of each lysin (Figure 3c, Table 3). This band was less strong for lysin Pa13. A preliminary assay, in which 30 µg of the lysin was applied in a bacterial lawn of *Micrococcus luteus,* showed that all expressed lysins produced growth inhibition halos (Figure 3d). Thus, it was shown that these four putative lysins are *de facto* peptidoglycan-hydrolysing lysins. All lysins retained their activity over one month at 4 °C. Only Pa7 and Pa119 were further considered for subsequent analysis.

### 2.4. Pa7 and Pa119 Have Peptidoglycan Hydrolase Activity

**Figure 4:**
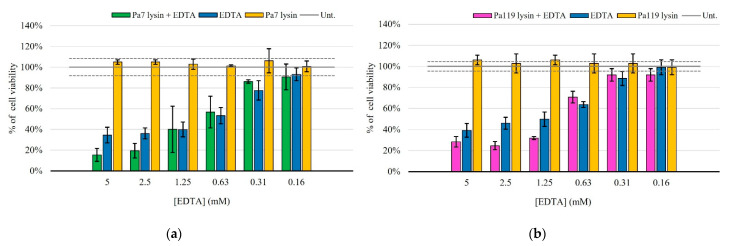
Antibacterial activity of lysins Pa7 (**a**) and Pa119 (**b**) in combination with the outer membrane permeabilizer EDTA in *P. aeruginosa* ATCC 27853. Results are shown as % of cell viability upon treatment and are based on three independent assays. Green—Pa7 lysin in combination with EDTA; pink—Pa119 lysin in combination with EDTA; blue—EDTA only; yellow—each lysin (Pa7 (**a**) or Pa119 (**b**)) applied solely. Untreated control is represented as a solid gray line, with the respective 95% confidence interval represented as dashed gray lines.

Gram-negative bacteria are resistant to many agents due to the highly efficient selective permeability of the outer membrane. This membrane is impermeable to macromolecules, allowing only a limited group of hydrophilic substances to diffuse through porins. For the integrity of the outer membrane contributes the negative charged lipopolysaccharide (LPS). This negative charge causes LPS to bind to cations, such as Ca^2+^ and Mg^2+^ [17]. Therefore, we used the membrane permeabilizer EDTA alone or in combination with the lysins Pa7 or Pa119. EDTA is a chelating agent, which, by binding to these cations, prevents their binding to the LPS present in the bacterial membrane, leading to the release of a significant proportion of the LPS from the outer cell membrane, thus, providing a facilitated access of lysins to the peptidoglycan. Our results showed that EDTA individually killed *P. aeruginosa*, while the endolysins under study, in the absence of this permeabilizer, had no effect on the bacterium. However, the synergy of both endolysins with EDTA was evident, especially at higher permeabilizer concentrations (Figure 4).

### 2.5. Liposome Deliver Phage Lysins to the Peptidoglycan of P. aeruginosa

Lysins were incorporated in a liposomal formulation composed of DPPC: DOPE: CHEMS (molar ratio 4:4:2). The rational for selecting this lipid composition is based on their fusogenic properties, due to the presence of DOPE and CHEMS that provide increased fluidity to the lipid bilayer, and may destabilize biological membranes [24,25]. Lysins Pa7 and Pa119 were efficiently incorporated in liposomes using this lipid composition and obtained physicochemical properties of developed nano formulation are shown in Table 4.

Encapsulation efficiencies of 33.33% and 32.30% for Pa7 and Pa119, respectively, were achieved. Both formulations displayed reduced mean sizes (<200 nm) with high homogeneity, demonstrated by a low polydispersity index (PI; <0.15). A negative zeta potential was obtained for all nano formulations due to the presence of CHEMS in the lipid composition.

**Figure 5:**
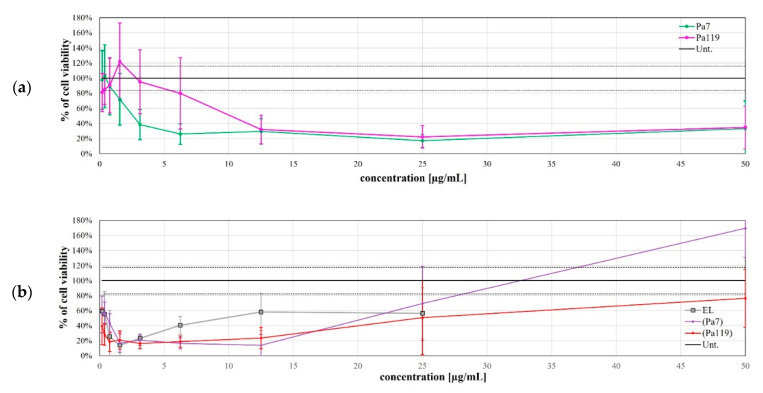
The bactericidal effect of the free (**a**) and encapsulated lysins (**b**) at 72 h post-treatment. The viability curve for the most effective time point (72 h p.t.) is shown. EL—empty liposomes; Pa7—free lysin Pa7 not encapsulated in liposomes; Pa119—free lysin Pa119 not encapsulated in liposomes; (Pa7)—encapsulated lysin Pa7 in liposomes; (Pa119)—encapsulated lysin Pa119 in liposomes; Unt.—untreated control. For both free lysins and encapsulated lysins, concentration refer to protein concentration applied; for empty formulation, concentration refers to the lipidic concentration tested to match the one used on encapsulated lysin application. The most effective concentration of the free lysins (**a**) is pointed with an orange arrow (25 µg/mL). The same is presented for encapsulated lysins (**b**) taking in consideration the effect of the empty formulations (6.25 µg/mL). The untreated control is shown as a black line, with the dashed lines representing the 95% confidence interval.

The ability of these formulations in delivering the encapsulated lysin to the peptidoglycan was tested in vitro using a *P. aeruginosa* strain as the model. Using an MTT assay approach to assess bacterial cell viability [39], the bactericidal effect of the free and encapsulated lysins upon application against *P. aeruginosa* was evaluated at several time-points. The most effective time-point and lysin concentration was determined for both lysins when applied as free lysin (Figure 5a) or encapsulated in liposomes (Figure 5b). The Pa7 and Pa119 free lysins were effective at 25 µg/mL (Figure 5a) and their encapsulated counterparts at 6.25 µg/mL (Figure 5b). The bactericidal effect of the encapsulated lysin did not strictly increase with the increasing amount of formulation applied. For instance, the application of over 25 µg/mL of any of the encapsulated lysins resulted in an increase in bacterial growth, even above the untreated control, suggesting a potential nutritional role of the lipids or trehalose used in buffer in the short term after application.

**Figure 6:**
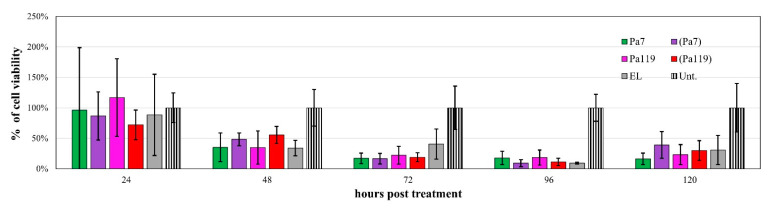
The bactericidal effect of the most effective concentrations of Pa7 and Pa119 up to 120 h post-treatment. EL—empty liposomes; Pa7—Pa7 lysin in the free form; Pa119—Pa119 lysin in the free form; (Pa7)—Pa7 lysin encapsulated in liposomes; (Pa119)—Pa119 lysin encapsulated in liposomes; Unt.—untreated control. The cell viability after treatment up to 120 h is shown for the most effective concentrations of lysins in the free form (25 µg/mL) and for lysins encapsulated in liposomes (6.25 µg/mL). The represented empty liposomes (EL) formulation bars correspond to the lipidic concentration used to match the same conditions of the encapsulated lysins. The untreated control is shown as a striped bar for comparison.

**Figure 7:**
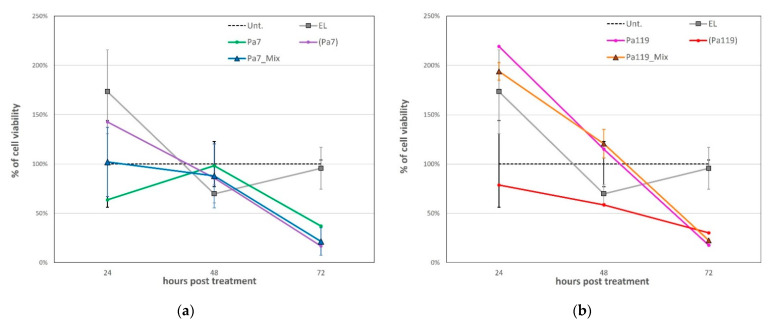
The bactericidal effect of the cocktail approach for lysins Pa7 (**a**) and Pa119 (**b**) and respective controls up to 72 h post-treatment. EL—empty liposomes; Pa7—Pa7 lysin in the free form; Pa119—Pa119 lysin in the free form; (Pa7)—Pa7 lysin encapsulated in liposomes; (Pa119)—Pa119 lysin encapsulated in liposomes; Pa7_Mix—lysin cocktail comprising free Pa7 lysin and Pa7 lysin encapsulated in liposomes; Pa119_Mix—lysin cocktail comprising free Pa119 lysin and Pa119 lysin encapsulated in liposomes; Unt.—untreated control. The untreated control is presented as a black dashed line for comparison. The combined application of free and encapsulated lysins did not increase their bactericidal effect.

The most effective time point before the toxic effect of the empty liposomes was 72 h post-application for both lysins (Figure 5 and Figure 6). Of note, the empty liposomes have a considerable negative impact on the bacterial viability per se, probably due to membrane destabilization. For lysins both in free and liposomal forms, a substantial reduction on bacterial viability was observed 48 h post treatment (Figure 6). Considering the most effective concentrations for lysins in free and liposomal forms, we tested a mix approach by applying a cocktail of each lysin in free and liposomal forms at the respective optimal concentration (Figure 7).

**Figure 8:**
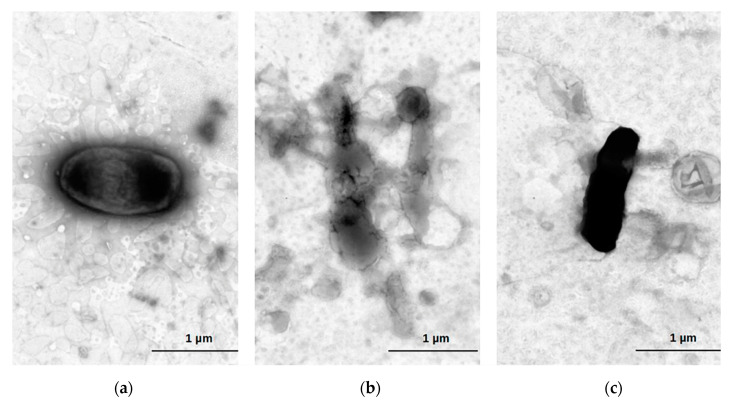
Cell death after exposure to liposome encapsuled lysins. Electron microscopy of *P. aeruginosa* ATCC 27853 treated with lysins encapsulated in liposomes. Untreated *P. aeruginosa* (**a**); *P. aeruginosa* treated with 100 μg/mL lysin Pa7 (**b**) and lysin Pa119 (**c**) after 120 h. Cells were negatively stained using 1% aqueous uranyl acetate.

Despite the hypothesis, the combination of each lysin in free and liposomal forms did not result in a higher decrease in the bacterial viability, at least when considering a single application. The negative staining micrographs showed lysed cells after exposition to encapsulated lysins Pa7 or Pa119, without a conserved cellular contour, while cells that were not exposed presented conserved cell wall structure, compatible with cell viability (Figure 8).

## 3. Discussion

Prophages change the genomic content of the host bacterium, sometimes providing an increased host fitness and virulence that impacts the evolution of their host species [31,40]. Our group of *P. aeruginosa* sequenced strains have complete prophages in 87.5% (14/16) of the cases. On average, these strains harboured two prophages within their genome. Such high prophage prevalence appears to be common to *P. aeruginosa* [41]. To increase the prophage diversity of our sample we have also included prophages described by others [27] or identified by us in genomes available in public databases. The phylogenetic analysis of a group of 38 prophages revealed a high diversity. Furthermore, the prophages newly identified here had no homology with the characterized ones, representing a considerable pool of new sequences for lysin hunting. During prolonged lysogeny, prophages may become remnant as a consequence of complex decay processes [42]. Remnant prophages are characterized by their incompleteness, where phage genes have been lost. To increase the chances of success in finding lysin genes, the analysis was restricted to complete prophages identified. Thus, this set of 38 prophages do not fully describe the complete prophage content in the analysed genomes, nor does it mean that other lysin gene may eventually be present in those remnant prophages. Prophages have been coevolving with bacteria for more than a billion years, during which an arms race allowed them to develop efficient strategies to lyse the host bacteria with consequent release of the phage progeny [31]. Lysin therapy assays to use the phage lytic protein as an enzybiotic, i.e., an enzyme-based antibacterial, against the bacterium that its prophage infects. Among the 38 prophages we were able to identify 19 phage lysins, found to be diverse in terms of nucleotide and amino acid sequences, predicted structure, and mode of action. Lysins lyse the host cell by targeting linkages that are essential for peptidoglycan integrity, meaning that although the target bond may be distinct, the outcome is identical, resulting in cell death [43,44]. The predicted mode of action of the lysins found in *P. aeruginosa* prophages is in agreement with that of lysins described for *Pseudomonas* phages [44]. Additionally, the distinct types of lysins found denote the mosaic nature of prophages, best explained by gene exchanges, and highlighted by the conflict between the topologies of the genome and lysin gene trees. Indeed, gene loss and gain, as well as recombinational exchanges of genes are very pervasive in phages [45,46]. The selected lysins had activity, hydrolysing the peptidoglycan and showing that even in the absence of a certainty that the prophage will follow a lytic cycle, these genes, once expressed, continue to lead to the translation of an active protein with catalytic activity on the peptidoglycan.

The cell lysis after phage infection consists of a lysis from within the cell, while in the biomedical application of lysins, the aim is to achieve cell lysis from without, by the application of lysins extracellularly [47]. The therapeutic use of lysins from the outside of the bacterial cell is facilitated among the Gram-positive bacteria, since lysins can make direct contact with the cell wall carbohydrates and peptidoglycan when added externally, whereas the outer membrane of Gram-negatives difficult this interaction [48]. In the Gram-negative *P. aeruginosa*, the use of outer membrane permeabilizer EDTA allowed to verify the cell lysis ability of the two identified lysins, Pa7 and Pa119. In this study, we found that, in conjugation with 5 mM EDTA, 100 µg/mL Pa7 and Pa119 reduced about seven times the OD in 60 min when incubated with *P. aeruginosa* cells. This result is in line with the application of lysins isolated from *Pseudomonas* phages, LysPA26 [49], EL188 [50], Ppl65 [51], and Ply17 [52], which presented activity against *P. aeruginosa* in the presence of EDTA. Only the lysin Ppl65 may derive from a phage with lysogenic capacity, since the *Pseudomonas* phage PPpW-3 (NC_023006.1) that codes for this lysin harbours an integrase gene. The strategy of overcoming the outer membrane with the use of EDTA encounters limitations, since EDTA inhibits blood coagulation, which is not suitable to treat systemic infections but is still useful for the treatment of topical *P. aeruginosa* infections, such as burn wound, eye, and ear infections [50]. Currently, EDTA is used as a medication in the treatment of heavy metal toxicity [53].

The major advantage of using liposomes against Gram-negative bacteria relies on their intrinsic ability to fuse with the outer membrane enabling the release of the transported drug [20], as well as their ability to treat systemic infections. In addition, liposomes as carrier systems are classified as safe and have demonstrated therapeutic versatility [54]. In this study, we showed that Pa7 and Pa119 maintain the lytic activity after encapsulation in the liposomal formulation, with an encapsulation efficiency of about 33%. The encapsulated lysins Pa7 and Pa119 in liposomes with the composition DPPC: DOPE: CHEMS (ratio 4:4:2) were able to kill *P. aeruginosa* in a concentration of 6.25 µg/mL, being the most effective time point achieved at 48 h after application. Of note, the free (not encapsulated lysins Pa7 and Pa119) showed some capacity to kill *P. aeruginosa*, even when at a higher concentration (25 µg/mL). These results showed that the chosen liposomal formulation is suitable for lysin delivery to the *P. aeruginosa* model, although the time taken to kill suggests that the fusion of the liposome with the outer membrane and/or the release of the lysin at peptidoglycan site is a gradual and slow process. It should be noted that there is no evidence for *P. aeruginosa* species specificity of the described liposomal lysin delivery system. Recently, the encapsulated lysin BSP16Lys (from the *Salmonella* phage BSP16) in liposomal formulation of dipalmitoyl phosphatidyl choline, cholesterol, and hexadecylamine (DPPC:Chol:HDA, at a ratio of 8:2:1) was able to kill the Gram-negative *Salmonella* Typhimurium and *E. coli* cells, presenting a similar bacterial load reduction of 2-log CFU/mL and 1.6-log CFU/mL for *S*. Typhumurium and *E. coli*, respectively [55]. Apart from that, liposomes (formulated of l-alpha-lecithin and sodium cholate in a ratio of 5:1; or l-alpha-lecithin and PEG2000 PE in ration of 10:1) were also able to deliver lysin to the Gram-positive *Streptococcus pneumoniae* [56]. Importantly, the treatment of *P. aeruginosa* infection in patients with cystic fibrosis relies on the use of inhaled antibiotic treatments that directly target the lungs. A major disadvantage is the fast clearance of the antibiotic from the lungs, allowing bacteria regrowth. However, liposomes may provide a sustainable and continuous delivery [57], as is the case of the liposomal amikacin, an inhalation therapy that reduces *P. aeruginosa* load in cystic fibrosis patients [58]. The pharmaceutical behaviour of encapsulated lysins in terms of pharmacokinetics, pharmacodynamics, and toxicity is to be characterized. We anticipate that, as with any protein drug, a limitation could be the production of antibodies that could hamper the repetitive uses of the lysin. However, the limitation by antibodies production may be overcome in liposomal delivery. Future work includes the determination of these formulations specificity, testing them against other Gram-negative species; in vitro toxicity assessment using human cell cultures exposed to the liposomal formulations; and in vivo studies to evaluate the potential of the liposomal formulations to treat *P. aeruginosa* infection. 

## 4. Materials and Methods

### 4.1. P. aeruginosa Genomes

A set of 16 *P. aeruginosa* isolates (samples ERS12519887 to ERS12519902 included in project accession PRJEB55011), collected between 1980 and 2004, of colonized or infected patients with *P. aeruginosa*, hospitalized at two Lisbon hospitals (Santo António dos Capuchos and Santa Maria) and from Setúbal Hospital (São Bernardo), were newly sequenced at the National Institute of Health (Lisbon, Portugal) using 2  ×  250 bp paired-end sequencing (Illumina MiSeq, Illumina Inc., San Diego, CA, USA), according to the manufacturer’s instructions. The FastQC (http://www.bioinformatics.babraham.ac.uk/projects/fastqc/) (accessed on October 2019) and FASTX (http://hannonlab.cshl.edu/fastx_toolkit/) (accessed on October 2019) tools were applied to evaluate and improve the quality of the raw sequence data, respectively. Genomes were *de novo* assembled using SPADES v13.3 [59]; and annotated using the Rapid Annotation using Subsystem Technology (RAST) [60].

Additionally, 50 genomes of *P. aeruginosa* obtained from PATRIC website (https://www.patricbrc.org) (last accessed January 2021) [28] were selected according to four criteria: isolates from the human host, country of isolation, date of collection, and MultiLocus Sequence Typing (MLST).

### 4.2. Prophage Identification and Classification

Prophages were screened as previously described [61]. Briefly, the prophage identification tools PHASTER (PHAge Search Tool Enhanced Release) [62] and Prophage Hunter Tool [63] were used for putative prophage identification and annotation in the group of 66 *P. aeruginosa* genomes. For our analysis purpose, only intact (according to PHASTER) and active (according to Phage Hunter) prophages were considered. Additionally, we have also included 3 prophage genomes (41Z; 42argF; 44G) described by Mageeney et al. (2020) in the analysis [27].

The identified complete prophages were classified in silico into their respective phage families based on the prophage structural head-neck-tail proteins using VIRFAM [26].

### 4.3. Endolysin Identification

Intact prophage sequences were submitted to bioinformatics analysis for identification of putative phage endolysins or related lytic enzymes in terms of sequence homology using Nucleotide Basic Local Alignment Search Tool (BLASTn) [64] and structural homology using the open access tools Phyre2 [35] and InterPro [34]. Endolysin genomic and proteomic sequences were aligned using multiple alignment using fast Fourier transform (MAFFT) (version 7.450) [65]. Poor alignment sequences were excluded using the bioinformatic tool Phylemon 2.0 [66]. The genome phylogenetic tree was constructed using the Jukes–Cantor substitution model and the proteome phylogenetic tree was constructed using the Le Gascuel substitution model in PHYML 3.3.20180621 (Geneious version 2021.1.1). Trees were visualized and annotated using Interactive Tree of Life (iTOL) v6 [30]. The most promising lysins candidates were selected with identity <20% and Phyre2 confidence prediction >90%, which allowed the selection of five potential endolysins for further study.

### 4.4. Lysin Genes Cloning 

The five putative lysins were chosen for cloning aiming to amplify sequence diversity and predicted biochemical lysin classification. These endolysins were codon-optimized for expression in *E. coli* BL21 (DE3), synthesized and cloned on the commercial vector pET15b (5708 bp) (Synbio Technologies, Aurora, CO, USA), with a resistance mark for ampicillin, and a *N*-terminal (His)^6^-tag. *E. coli* BL21 (DE3) cells were transformed with 0.06 µg of construct by heat-shock procedure, as described previously [67]. The constructs were confirmed by colony PCR using GoTaq^®^ DNA Polymerase (Promega, Madison, WI, USA) and T7 promoter and T7 terminator primers (STABVida, Lisbon, Portugal), with standard PCR program, followed by Sanger sequencing (STABVida, Lisbon, Portugal).

### 4.5. Protein Expression and Purification

Single colonies of *E. coli* BL21(DE3) transformed with the pET15b-cloned putative endolysin sequence were grown overnight at 37 °C in LB broth supplemented with 100 µg/mL ampicillin (Sigma-Aldrich, St. Louis, MO, USA), under strong agitation, and used as pre-inoculum in new LB broth for expression of the putative endolysins. The bacterial cultures were grown in LB broth supplemented with 100 µg/mL ampicillin (Sigma-Aldrich) at 37 °C under strong agitation, up to an OD_600nm_ ≈ 0.6 and the expression of the putative endolysins was induced with Isopropyl β-D-1-thiogalactopyranoside (IPTG) for 3 h. Induced cultures were ice-cooled and then centrifuged (3220× *g*, 10 min, 4 °C) for cell recovery. The pellet was washed with Tris-HCl 20 mM pH 8.0 and centrifuged again under the same conditions. The supernatant was then totally removed, and the pellets were frozen at −20 °C for short term processing or −80 °C for long term storage. In the case of absence of expression, different IPTG concentrations were tested (0.1, 0.4 and 1 mM) and expression at lower temperatures (18 °C) was assayed, keeping the remaining procedure, but extending the induction time for 20 h. Induction confirmation was performed using sodium dodecyl sulfate polyacrylamide gel electrophoresis (SDS-PAGE) [68] and western blot [69].

Small scale preliminary purifications were performed using Ni-NTA Spin kit columns (Qiagen, Hilden, Germany) following manufacturer’s instructions. Buffer exchange was performed using Vivaspin 500 centrifugal concentrators (Sigma-Aldrich), with a 5000 Da molecular weight cut-off, following manufacturer’s instructions or using PD-10 desalting columns (GE Healthcare Life Science, Chicago, IL, USA), following manufacturer’s instructions, using PBS 1/15X with Trehalose (for a final osmolarity of 100 mOsm, PBS-Trehalose) as final buffer. In up-scaled purifications, the Ni-NTA agarose resin purification (Qiagen), or *n*—small scale purifications were performed following manufacturer’s instructions. Buffer exchange using PBS-Trehalose was done using PD-10 columns (Cytiva, Marlborough, MA, USA).

The purified endolysins were quantified in Qubit fluorometer (Invitrogen, Carlsbad, CA, USA), and confirmation of protein purification and size was done by 12% SDS-PAGE. Additionally, to confirm the expression of the desired protein, a western blot was done using as primary antibody the His-tag (CT) rabbit polyclonal antibody (Bioss, Woburn, MA, USA) and the Goat Anti-Rabbit IgG (H+L)-HRP Conjugated antibody (Bio-Rad, Berkeley, CA, USA) in PBS-Tween +1% BSA, as secondary antibody.

### 4.6. Zymogram Analysis

Zymogram assay for detection of bacteriolytic activity of the putative lysins were performed as previously described (2011) [70], with adjustments considering the use of Gram-negatives that have thinner layer of peptidoglycan. For cell preparation, *M. luteus* and *P. aeruginosa* were grown in LB Broth, at 37 °C, until stationary phase. Cells were collected, suspended in water, and then autoclaved. The autoclaved cells were resuspended in water to a final concentration 2% (dry weight). For the Gram-positive *M. luteus*, 0.2% cell preparation, and for the Gram-negative bacteria *P. aeruginosa*, 0.4% cell preparation were incorporated on the 12% polyacrylamide gel. After electrophoresis, the zymogram gels were incubated in a renaturation buffer (25 mM Tris-HCl pH 7.5 and 1% Triton X-100) overnight at 37 °C, with mild agitation. The gels were stained for 90 min in zymogram staining solution (0.5% Methylene Blue and 0.01% KOH) and distained in distilled water. Lysozyme of egg white (1 µg) (Sigma-Aldrich, St. Louis, MO, USA) and BSA (5 µg) (NZYTech, Lisbon, Portugal) were used as positive and negative control, respectively. For migration control, a regular SDS-PAGE was performed in parallel and stained with BlueSafe (NZYTech, Lisbon, Portugal).

### 4.7. Antimicrobial Activity of Endolysins

For preliminary lysin activity tests, Gram-positive *M. luteus* bacteria were used. A *M. luteus* suspension was plated in LB-agar plates by flooding. Half-centimeter or one-centimeter round wells (respectively, with 75 µL and 200 µL of capacity) were made in the plates and used as application spots for 30 µg of the putative endolysins. Loaded plates were incubated at 37 °C for 48 h. Bacteriolytic effect was qualitatively assessed by the presence or absence of inhibition growth halos around the wells. Pre-obtained active endolysins and protein elution buffers were included as positive and negative controls, respectively.

Antibacterial activity in combination with the outer membrane permeabilizer EDTA was analyzed for stationary phase of the model organism *P. aeruginosa* ATTCC 27853, resuspended in 20 mM Tris-HCl for an OD_600nm_ adjusted to 1.2. A series of dilutions by 1:1 starting with 5 mM of EDTA was used. The endolysin was applied to a final concentration of 100 µg/mL. PBS-Trehalose was used as negative control. Plates were incubated at 37 °C, with agitation and the OD_600nm_ was measured after 1 h. All assays were performed in triplicate.

### 4.8. Minimum Inhibitory Concentration (MIC) and Minimum Bactericidal Concentration Determination (MBC)

The minimum inhibitory concentration (MIC) and minimum bactericidal concentration (MBC) of the up-scale purified putative lysins were assessed by serial dilutions method [71]. The first well of a 12-well plate contained 100 µg/mL of the tested putative lysin and the following wells were made by 1:1 serial dilutions in LB broth. Each well was then inoculated with 20 µL of a *M. luteus* suspension (OD_600nm_ = 0.4). Positive and negative controls were included. After 24 h of incubation, at 37 °C with agitation, the optical density (OD_600nm_) of each well suspension was measured in Varioskan LUX Multimode Microplate Reader (Thermo Scientific, Waltham, MA, USA). The MIC was the lowest putative lysin concentration at which the *M. luteus* visible growth was inhibited. After, to determine the MBC, 4 µL of each well suspension were spotted in LB agar plates and incubated overnight at 37 °C. The MBC was the lowest putative lysin concentration at which no growth was observed in the solid medium.

### 4.9. Encapsulation of Selected Endolysins in Liposomes 

Encapsulation of the purified putative endolysins in liposomes and their characterization was performed. Briefly, liposomes composed of dioleoyl phosphatidylethanolamine (DOPE), dipalmitoyl phosphatidyl choline (DPPC) and cholesteryl hemisuccinate (CHEMS) at a molar ratio of 4:4:2 were used. Liposomes were prepared by dehydration-rehydration method, followed by an extrusion step to reduce, and homogenize the vesicles size, as described previously [72]. The selected phospholipids were dissolved in chloroform and the solvent was evaporated (Buchi R-200 rotary evaporator, Switzerland) to obtain a thin lipid film in a round-bottomed flask. The lipid film was then dispersed with a lysin aqueous solution (500–850 µg/mL), and the so-formed suspension was frozen (−70 °C) and lyophilized (freeze-dryer, Edwards, CO, USA) overnight. The rehydration of the lyophilized powder was performed in phosphate-buffered saline, pH 7.4, (PBS) in two steps, to enhance the lysin incorporation [73]. First, a volume up to one third of the initial dispersion volume was added. After 30 min, PBS was added up to the original volume. The rehydration process was performed above the phase transition temperature (Tc) of the main phospholipid (+41 °C). The so-formed liposomal suspensions were then filtered, under nitrogen pressure (10–500 lb./in^2^), through polycarbonate membranes of appropriate pore size until an average vesicle size of 150 nm was obtained, using an extruder device (Lipex: Biomembranes Inc., Canada). The separation of non-incorporated lysin was performed by ultracentrifugation at 250,000× *g*, for 120 min, at 15 °C in a Beckman LM-80 ultracentrifuge (Beckman Instruments, Inc., Fullerton, CA, USA). Finally, the pellet was suspended in PBS. Unloaded liposomes were also prepared with the same lipid composition.

Endolysin liposomal formulations were characterized in terms of lipid and protein content and by the following incorporation parameters: initial and final lysin to lipid ratios ((Lysin/Lip)i and (Lysin/Lip)f, respectively). The encapsulation efficiency (E.E.) was defined as a percentage:

E.E. = [(Lysin/Lip)f]/[(Lysin/Lip)i] × 100

Lysin concentration incorporated in liposomes was determined using the Lowry method [74] with previous disruption of the vesicles with Triton X-100 and sodium dodecyl sulfate (SDS) [75]. Samples, in triplicate, containing a lysin amount between 7 and 35 µg (maximum volume, 0.5 mL) were used. A calibration curve with bovine serum albumin (BSA) was used. BSA standards ranged from 5 to 40 µg. The lipid content was determined using an enzyme-linked colorimetric method, phospholipids choline oxidase-peroxidase (Spinreact, SA, Sant Esteve de Bas, GI, Spain).

Liposomes mean size was determined by dynamic light scattering using Zetasizer Nano S (Malvern Instruments, Malvern, UK) at a standard laser wavelength of 663 nm. The system also reports a polydispersity index as a measure of particle size distribution, ranging from 0.0, for an entirely monodisperse sample, up to 1.0, for a polydisperse suspension. Zeta potential of liposomal formulations was measured in a hydrodynamic sizing system using Zetasizer Nano Z (Malvern Instruments, Malvern, UK).

### 4.10. Antimicrobial Activity of Encapsulated Endolysins

For assessing the bactericidal effect of the different lysins and formulations, MTT assays were conducted using a *P. aeruginosa* strain as model organism. This organism model was chosen because the newly identified lysins were retrieved from *P. aeruginosa* prophages. These assays take advantage of the metabolic active cell-mediated reduction of the yellow tetrazolium MTT salt, originating purple-blue crystals of formazan that can be solubilized and quantified by spectrophotometry [39]. Briefly, serial dilutions (1:1) of the different compounds to test were made in 24-well plates to a final volume of 1 mL/well of PBS-Trehalose. An overnight pre-inoculum of the model organism *P. aeruginosa* ATCC 27833 was inoculated in LB broth to an OD_600nm_ ≈ 0.2 and let to grow up to an OD_600nm_ ≈ 1.0 at 37 °C, under strong agitation. The grown culture was centrifuged, and the supernatant discarded. The pelleted bacteria were resuspended in the same volume of PBS-Trehalose, and 50 µL of this suspension were used for inoculation in the previously prepared 24-well plates. The inoculated plates were incubated at 37 °C, with agitation, up to 120 h. At each time point, 100 µL of each well’s suspension were transferred to 96-well plates. To avoid the impact of the liposomes on the following colorimetric reaction, the plates were centrifuged (3220× *g*, 10 min, at room-temperature) and the supernatant discarded. The precipitate was resuspended in 100 µL of PBS-Trehalose, followed by the addition of 10 µL of MTT Cell Viability Assay Kit (Biotium, Fremont, CA, USA) and incubation for 3 h, at 37 °C, with agitation. After the incubation, 200 µL of dimethyl sulfoxide (DMSO) (Sigma-Aldrich) were added to each well to dissolve the formazan crystals and the absorbance was measured with a Varioskan LUX Multimode Microplate Reader (Thermo Fisher Scientific, Waltham, MA, USA) at 570 nm, with background at 630 nm, according to manufacturer instructions. Aliquots of the highest lysin concentration were picked for observation by transmission electron microscopy (JEOL 100SX) after negative staining, as previously described [12,76].

### 4.11. Cocktail Assays

After identifying the most effective concentrations of the free (25 µg/mL) and encapsulated lysins (6.25 µg/mL), a mixed approach was tested by applying a cocktail of free and encapsulated lysin at the referred concentrations to the model organism *P. aeruginosa* ATCC 27833. The assays were conducted in 24-well plates, in a final volume of 1 mL/well of PBS-Trehalose, inoculated with 50 µL of a *P. aeruginosa* ATCC 27833 suspension prepared as described above. The 24-well plates were incubated at 37 °C with agitation, up to 72 h, and MTT assays to assess the bactericidal effect of the cocktail were performed at each time point, as described above.

## 5. Conclusions

We showed here for the first time that *P. aeruginosa* prophages constitute an invaluable source of lysins with activity against this bacterium, avoiding the need of phage isolation. We have shown here that a search for prophages and their lytic enzymes constitutes a good source of lysins with therapeutic potential against *P. aeruginosa.* Moreover, these lysins were successfully expressed and encapsulated within liposomes with a reasonable efficiency. We have shown here for the first time that liposome encapsulated lysins kill *P. aeruginosa* without pre-treatment with an outer membrane permeabilizer. Although further investigation and additional experiments are needed to increase the efficacy of Pa7 and Pa119 and comprehend the mechanism of lysin delivery by liposomes, this strategy of lysin delivery to *P. aeruginosa* is highly promising and may contribute to the fight against antibiotic-resistant bacteria.

## Figures and Tables

**Table 1:** Genomic characterization of the complete prophages.

Prophage	GC(%)	Size (kb)	CDS	Coding Region (%)	tRNA	Family	Reference
41Z	60.60	40.94	70	92.57	0	*Siphoviridae*	[27]
42argF	61.80	41.59	67	92.92	0	*Siphoviridae*	[27]
44G	63.00	43.67	54	90.81	0	*Siphoviridae*	[27]
L1378_17	64.20	28.58	36	91.26	0	*Myoviridae*	This study
L1378_56	57.30	38.16	50	86.99	3	*Siphoviridae*	This study
L1379_42	62.00	37.57	51	90.39	0	*Myoviridae*	This study
L1395_55	58.70	35.27	60	81.40	2	ND	This study
L1395_78	60.00	21.74	25	84.85	0	*Siphoviridae*	This study
L3941_30	62.50	31.45	38	89.85	0	*Myoviridae*	This study
L3942_9	61.10	62.09	84	82.46	0	*Siphoviridae*	This study
L3944_13	60.80	34.57	50	91.42	0	*Siphoviridae*	This study
L3944_48	58.80	56.82	101	77.15	0	ND	This study
L3944_59	59.90	45.34	68	88.79	0	*Siphoviridae*	This study
L3945_7	58.90	33.76	42	77.92	0	*Siphoviridae*	This study
L3946_2	64.40	37.21	57	95.86	0	*Siphoviridae*	This study
L3946_67	57.30	30.85	42	89.90	3	ND	This study
L3955_1	60.20	37.76	48	87.82	0	*Myoviridae*	This study
L3955_10	62.70	36.34	45	88.47	0	*Myoviridae*	This study
L3955_30	58.90	53.78	87	86.32	1	*Siphoviridae*	This study
L4112_2	57.80	49.05	72	85.93	2	*Siphoviridae*	This study
L4112_49	62.70	42.67	53	87.04	0	*Siphoviridae*	This study
L4137_3	62.00	66.16	65	90.81	0	*Podoviridae*	This study
L4139_13	62.70	39.72	55	95.00	0	*Siphoviridae*	This study
L4140_27	58.70	28.55	53	82.15	1	ND	This study
L4140_48	63.90	29.22	44	88.65	0	ND	This study
L4441_19	60.80	74.64	77	84.36	0	ND	This study
L4441_52	62.20	38.28	46	87.18	0	*Siphoviridae*	This study
O4_56090	64.20	37.18	55	95.37	0	*Siphoviridae*	[28]
O5_46060	59.80	58.78	45	86.48	0	ND	[28]
O5_46403	55.40	35.8	34	88.88	0	ND	[28]
O8_56090	59.00	60.54	99	85.63	2	*Siphoviridae*	[28]
O1_56090	61.70	40.52	62	93.48	0	*Siphoviridae*	[28]
O4_16214	60.30	48.74	53	74.68	1	*Siphoviridae*	[28]
O3_43328	63.40	38.77	53	95.02	0	*Siphoviridae*	[28]
O5_61780	62.30	39.78	60	93.77	0	*Siphoviridae*	[28]
O7_12585	61.90	72.9	69	93.46	0	*Siphoviridae*	[28]
O8_43328	64.60	57.23	66	92.92	0	*Podoviridae*	[28]
O8_61780	62.10	53.39	72	85.91	0	*Siphoviridae*	[28]

ND—not determined. Phage from newly sequenced genomes start with letter L; from PATRIC database start with O, while the ones from the study of Mageeney et al. [27] use the attributed phage name (41Z and 42argF).

**Table 2:** Functional analysis of putative lysins inferred by protein sequence and predicted structure.

Putative Lysin	InterPro [34]		Phyre [35]
	Family	Description	Description
41Z_15	Repressor Cro	Helix-turn-helix	Chitinase
41Z_58	-	-	Endolysin
41Z_63	-	-	Endolysin
42argF_542 *	Phage lysis	Phage lytic protein Rz	Endopeptidase
L4112_2_7 *	Phage lysozyme	Lysozyme	Lysozyme
L3944_13 *	Glycosidic hydrolase family 19	Chitinase class I	Endolysin
L4139_13_15 *	Transglycosylase	Transglycosylase	Transglycosylase
L3955_10_119 *	Muramidase	*N*-acetylmuramidase	Peptidoglycan binding domain
L1378_17	Phage lysozyme	Lysozyme	Endolysin
L1395_55	-	-	Endolysin
L3941_30	Phage lysozyme	Lysozyme	Endolysin
L3944_48	-	-	Endolysin
L3944_482	-	-	Endolysin
L3946_2	-	-	Endolysin
L3946_22	-	-	Endolysin
L4112_2	Endopeptidase	Endopeptidase	Lysozyme
L4137_3	Phage lysozyme	Lysozyme	Lysozyme
L4140_27	-	-	Transglycosylase
O4_16214	Peptidase	Endopeptidase	Hydrolase

* Selected lysins for cloning. Phage lysins from newly sequenced genomes starting with letter L; from PATRIC database starting with O, while the ones from the study of Mageeney et al. [27] use the attributed phage name (41Z and 42argF).

**Table 3:** Selected lysins characterization.

Lysin	BLASTp [37]				Molecular Weight (kDa)		Theoretical pI [38]	Number of Aminoacids	Zymogram (Peptidoglycan Hydrolyzing Activity)	
	Classification	Query Cover (%)	Identity (%)	E-Value	Predicted Protparam [38]	Experimental SDS-PAGE			*M. luteus*	*P. aeruginosa*
Pa7	Lysozyme	100	99.31	1.00 × 10^−101^	16.20	16.5	9.69	144	yes	yes
Pa13	Chitinase	100	100	3.00 × 10^−151^	22.86	25.0	9.41	205	yes	yes
Pa15	Transglycosylase	100	98.56	6.00 × 10^−150^	23.50	18.5	9.54	209	yes	yes
Pa119	*N*-acetylmuramidase	99	99.25	0.00	29.30	30.0	7.13	268	yes	yes
Pa542	Rz lytic protein	99	95.97	8.00 × 10^−99^	16.06	ND	9.23	150	ND	ND

ND—not determined; pI—isoelectric point.

**Table 4:** Characterization of the liposomal formulations.

Lipid Composition (Molar Ratio)	Lysin	(Prot/Lip)i (μg/μmol)	(Prot/Lip)f (μg/μmol)	E.E. (%)	Mean Size (nm)	P. I.	Zeta Pot (mV)
DPPC:DOPE:CHEMS (4:4:2)	Pa7	16 ± 5	5 ± 2	33 ± 7	151 ± 6	0.111 ± 0.011	−21 ± 2
DPPC:DOPE:CHEMS (4:4:2)	Pa119	10 ± 3	3 ± 1	32 ±9	149 ± 4	0.106 ± 0.012	−22 ± 2
DPPC:DOPE:CHEMS (4:4:2)	EL	NA	NA	NA	142 ± 2	0.125 ± 0.015	−22 ± 1

Values are presented as average ± standard deviation, based on 6 to 9 independent experiments. NA—not applicable; Prot—protein; Lip—lipid; E.E.—encapsulation efficiency; P.I.—polydispersion index; EL—empty liposomes. Lysin Pa7 initial concentration ranged from 650 to 850 μg/mL. Lysin Pa119 initial concentration ranged from 500 to 750 μg/mL.

## Data Availability

Genome sequencing data are available at ENA under project accession PRJEB55011, samples ERS12519887 to ERS12519902.
